# Exposure to interparental violence and risk of intimate partner violence among women in sexual unions in sub-Saharan Africa

**DOI:** 10.1093/inthealth/ihac075

**Published:** 2022-12-02

**Authors:** Richard Gyan Aboagye, Abdul-Aziz Seidu, Prince Peprah, Bernard Yeboah-Asiamah Asare, Isaac Yeboah Addo, Bright Opoku Ahinkorah

**Affiliations:** Department of Family and Community Health, Fred N. Binka School of Public Health, University of Health and Allied Sciences, Ho, Ghana; Department of Population and Health, University of Cape Coast, Cape Coast, Ghana; College of Public Health, Medical and Veterinary Sciences, James Cook University, Australia; Centre for Gender and Advocacy, Takoradi Technical University, P.O. Box, 257, Takoradi, Ghana; Social Policy Research Centre, University of New South Wales, Sydney, Australia; Centre for Primary Health Care and Equity, University of New South Wales, Sydney, Australia; Curtin School of Population Health, Curtin University, Perth, Australia; Institute of Applied Health Sciences, University of Aberdeen, Aberdeen, Scotland, United Kingdom; Centre for Social Research in Health, University of New South Wales, Sydney, Australia; School of Public Health, Faculty of Health, University of Technology Sydney, Sydney 2052, NSW, Australia

**Keywords:** Interparental violence, intimate partner violence, sub-Saharan Africa, women

## Abstract

**Background:**

*Intimate partner violence (IPV) has emerged as a serious human rights issue as well as a public health concern globally*. We examined the association between exposure to interparental violence and experience of intimate partner violence (IPV) among women in sub-Saharan Africa (SSA) using Demographic and Health Survey (DHS) data.

**Methods:**

We included 96 782 women in 23 countries with a recent DHS dataset conducted from 2012 to 2020. We employed multivariable multinomial logistic regression to examine the association between exposure to interparental violence and experience of IPV.

**Results:**

Overall, women who were exposed to interparental violence were more likely to experience physical (adjusted OR [aOR]=2.17, 95% CI 2.07 to 2.28), emotional (aOR=1.87, 95% CI 1.78 to 1.96) and sexual violence (aOR=2.02, 95% CI 1.90 to 2.15) than those who were not exposed. In all countries except Comoros, women exposed to interparental violence had higher odds of physical and emotional violence than those who were not exposed. Experience of sexual violence was higher among women exposed to interparental violence from all countries except for Liberia, Rwanda and Sierra Leone.

**Conclusions:**

Women's exposure to interparental violence increases their risk of experiencing IPV. Policies should focus on women or young girls who who witness IPV to reduce their likelihood of experiencing it. Special support can be provided for women who were exposed to interparental violence and are currently experiencing IPV.

## Introduction

Intimate partner violence (IPV), which comprises a range of sexually, emotionally and physically coercive acts perpetrated in an intimate relationship context,^1^ has emerged as a serious human rights issue as well as a public health concern globally.^2–4^ Although both males and females can be IPV victims, the paternalistic culture of most developing countries, especially those in sub-Saharan Africa (SSA), encourages male dominance and exposes women to a higher risk of IPV.^2–5^ Globally, available statistics suggest that 1 in 3 (30%) of women have been subjected to either physical and/or sexual intimate partner violence or non-partner sexual violence in their lifetime,^6^ although the prevalence of IPV differs by country.^2,4,7^ Worldwide, almost one-third (27%) of women aged 15-49 y who have been in a relationship reported that they have been subjected to some form of physical and/or sexual violence by an intimate partner.^6^ Evidence also suggests that SSA has the highest prevalence of IPV, with an overall prevalence of 51.1%,^7^ which is higher than the global average of 30%.^1^ For example, a recent study of 16 countries found that IPV prevalence ranges from 44.0% in Uganda to 12.7% in South Africa.^2^ Another recent study conducted across 23 SSA countries found that IPV prevalence ranges from 51.1% in Sierra Leone to 7.4% in Comoros.^7^

The experience of IPV is accompanied by social and health consequences. Several studies have linked IPV to a variety of social and health consequences, including pregnancy loss after experiencing violence and the transmission of sexually transmitted infections.^1,8^ Furthermore, women who have experienced IPV are more likely to report psychological and mental health problems, such as depression, post-traumatic stress disorder and suicidal ideation.^1,9^

Given the alarming prevalence of IPV and the physical, emotional and psychological consequences for its victims, it is critical to identify and examine risk factors associated with such abuse. Evidence from previous studies have shown that women's experiences with IPV are linked to a complex set of individual, household, dyadic and societal level factors.^2,5,10^ Numerous studies in SSA have identified sociodemographic factors, such as age at first marriage, spousal age difference, education, wealth index and place of residence, among others, as predictors of IPV.^11–13^ Others have found polygyny to be a significant predictor of IPV.^2^ Identifying and addressing these risk factors can reduce both IPV prevalence and its consequences.^3^

However, there is evidence that other more specific and direct factors might serve as better determinants of IPV-specific prevention initiatives.^3^ Emerging evidence suggests that exposure to interparental violence is one of the significant psychological risk factors that can lead to the normalization of IPV in intimate relationships.^4,14,15^ The association between witnessing IPV in childhood and later perpetration has been found in previous studies.^16,17^ Witnessing interparental violence can affect children's physical and biological functioning, behavior, emotions, cognitive development and attitudes toward the use of violence and conflict resolution.^17^

To the best of our knowledge, there are few studies conducted in developing countries^3,4,18,19^ that have examined interparental violence and IPV. However, these studies did not explicitly explore and examine the relationship between exposure to interparental violence and the experience of IPV among women in sexual unions. For instance, the study by Islam et al.^3^ only focused on ever-married men, whereas the study by Abramsky et al.^4^ was less focused on associations between interparental violence and IPV. Interestingly, studies in Nigeria found that women who had ever witnessed interparental violence were more likely to be abused by their spouse than those who had not witnessed parental IPV.^19,20^

The gaps in the available studies suggest that there is a need for a multi-country study on the association between exposure to interparental violence and experience of IPV in developing countries, particularly in SSA. Based on this, the overall aim of this study was to examine the association between exposure to interparental violence and experience of IPV among women in SSA using Demographic and Health Survey (DHS) data from 23 countries. The findings from this study can inform the development of nuanced policies, programs and interventions aimed at addressing women's exposure and experience of interparental and IPV in SSA. This study is motivated by Abramsky et al.'s^4^ observation that developing effective IPV prevention programs requires identifying risk factors: both those that are direct causes of IPV and those that point to common characteristics of victims and/or perpetrators, allowing for appropriate tailoring and targeting of services. Thus, for intervention development, this study is being carried out to maximize the chances of IPV intervention success while minimizing the possibility of inadvertently harming victims of violence in SSA.

## Methods

### Data source and study design

We performed a cross-sectional analysis of DHS data from 23 countries in SSA. This study included countries that had information on DHS's domestic violence modules. The individual recode dataset (women's file) was used. The DHS is a nationally representative survey that collects data on a wide range of health indicators, including domestic violence, in >85 low- and middle-income countries.^21^ DHSs are usually carried out every 5 y.^21^ The DHS used a two-stage cluster sampling method. The first stage entails the selection of clusters known as enumeration areas. The second stage consists of selecting households for the survey. A previous study^22^ highlighted the detailed sampling technique and data collection procedure. We included countries with datasets from 2012 to 2020 in this study. Furthermore, only countries with complete cases of variables of interest were considered in the final analysis. The study included 96 782 women who are currently married or cohabiting. The countries included in this study are listed in Table 1. The dataset is freely accessible via this link: https://dhsprogram.com/data/available-datasets.cfm. We relied on the Strengthening Reporting of Observational Studies in Epidemiology (STROBE) reporting guidelines in drafting this paper.^23^

**Table 1.** Description of study sample

S/N/country	Time of survey	Weighted N	Weighted %
1. Angola	2015–2016	7620	7.9
2. Benin	2017–2018	4024	4.1
3. Burundi	2016–2017	6089	6.3
4. DR Congo	2013–2014	4643	4.8
5. Cameroon	2018	3920	4.0
6. Ethiopia	2016	4248	4.4
7. Gabon	2012	2730	2.8
8. Gambia	2013	3054	3.2
9. Kenya	2014	3559	3.7
10. Comoros	2012	2031	2.1
11. Liberia	2019–2020	1664	1.7
12. Mali	2018	3293	3.4
13. Malawi	2015–2016	4518	4.7
14. Nigeria	2018	8495	8.8
15. Namibia	2013	862	0.9
16. Rwanda	2014–2015	1520	1.6
17. Sierra Leone	2019	3579	3.7
18. Chad	2014–2015	3086	3.2
19. Togo	2013–2014	4680	4.8
20. Tanzania	2015–2016	6443	6.6
21. Uganda	2016	6058	6.3
22. Zambia	2018	5696	5.9
23. Zimbabwe	2015	4968	5.1
All countries		96 782	100.0

Abbreviation: DR Congo, Democratic Republic of Congo.

### Study variables

#### Outcome variables

The main outcome variables were past year experience of physical, emotional and sexual violence. The outcome variables were derived from the domestic violence model, where questions were based on a modified version of the conflict tactics scale.^24,25^ The specific questions used to assess each of the violence variables (physical, emotional and sexual) include:

- Physical violence: the respondents were asked whether their partner has ever: pushed, shook or thrown something at them; slapped her; punched her with his fist or something harmful; kicked or dragged her; strangled or burnt her; threatened her with a knife, gun or other weapons; and twisted her arm or pulled her hair.
- Emotional violence: with this variable, the respondents were asked whether their partner ever: humiliated her; threatened to harm her; and insulted or made her feel bad.
- Sexual violence: the respondents were asked whether their partner ever: physically forced the respondent into unwanted sex; whether the partner ever forced her into other unwanted sexual acts; and whether the respondent has been physically forced to perform sexual acts she did not want to.

To each of the questions, the response options were ‘never’, ‘often’, ‘sometimes’ and ‘yes, but not in the last 12 months’. The response options were further categorized into ‘No’ (those who responded ‘never’ and ‘yes, but not in the last 12 months’) and ‘Yes’ (those who responded ‘often’ and ‘sometimes’). Previous studies^2,7^ that utilised the DHS datasets used these questions and categorizations to measure physical, emotional and sexual violence.

### Key explanatory variable

Interparental violence is the main explanatory variable in the present study. To assess this variable, the women were asked the question “As far as you know, did your father ever beat your mother?”. The response options were 0=No; 1=Yes; and 8=Don't know. Those who responded ‘No’ and ‘Don't know’ were grouped as ‘Not exposed’, while those who responded ‘Yes’ were categorized as ‘Exposed’ to interparental violence. Similar coding was used in studies conducted in Nigeria^20^ and Bangladesh.^3^

### Covariates

A total of 11 covariates were studied. These variables consisted of maternal age (y), maternal educational level, marital status, maternal current working status, exposure to radio, exposure to television, exposure to newspapers/magazines, partner's age (y), partner's educational level, wealth index and place of residence. These variables were selected for inclusion into the study based on their significant association with IPV^3,20^ as well as their availability in the DHS datasets. During the recoding, we maintained the existing coding in the DHS for the educational levels for the respondent and the partner (no education, primary, secondary and higher), maternal current working status (yes/no), wealth index (poorest, poorer, middle, richer and richest) and place of residence (urban/rural). Marital status was recoded as ‘married’ and ‘cohabiting’. The partner's age was recoded as ‘15–24 y’, ‘25–34 y’, ‘35–44 y’ and ‘≥45 y’. Also, each of the exposure to radio, exposure to television, and exposure to newspaper/magazine was coded as ‘Yes’ and ‘No’.

### Statistical analyses

Data analyses were performed using Stata version 16.0 (Stata Corporation, College Station, TX, USA). We employed percentages to estimate the prevalence of interparental violence and past year experience of each of the violence variables (physical, emotional and sexual). The Pearson χ^2^ test of independence was used to determine the relationship between interparental violence exposure, studied covariates and experiences of physical, emotional and sexual violence (Table 2). Multivariable multinomial logistic regression was used to determine the association between interparental violence and physical, emotional and sexual violence. Results from the regression analysis were presented as adjusted odds ratios (aORs) with their respective 95% confidence intervals (CIs). The statistical significance level was set at p<0.05. We performed a multicollinearity test using the variance inflation factor (VIF). The minimum, maximum and mean VIFs were found to be 1.03, 6.88 and 2.73, respectively. As a result, there was no evidence of multicollinearity among the variables investigated. All missing data were dropped. The women's sample weights (d005/1 000 000) for the domestic violence variables were used to obtain unbiased estimates in accordance with DHS guidelines, and the survey command (svy) in Stata was used to adjust for the complex sampling structure of the data in all analyses.

**Table 2.** Prevalence of exposure to interparental violence and IPV among women in SSA

Country	Exposure to interparental violence (%)	Physical violence (%)	Emotional violence (%)	Sexual violence (%)
1. Angola	27.3	24.1	24.6	6.6
2. Benin	10.0	10.9	29.2	6.1
3. Burundi	40.3	19.0	17.3	20.3
4. DR Congo	33.1	29.7	28.5	19.7
5. Cameroon	21.4	19.2	22.6	6.6
6. Ethiopia	27.3	16.8	20.0	8.5
7. Gabon	33.7	28.7	25.8	11.0
8. Gambia	8.9	6.3	7.4	1.1
9. Kenya	36.4	22.3	23.8	9.8
10. Comoros	4.7	3.8	5.5	1.2
11. Liberia	23.4	35.8	36.4	7.3
12. Mali	10.3	17.9	28.0	7.8
13. Malawi	25.8	15.3	22.3	15.5
14. Nigeria	9.9	11.5	27.1	4.6
15. Namibia	24.8	17.3	19.9	5.9
16. Rwanda	38.4	17.3	18.7	8.4
17. Sierra Leone	27.9	39.0	39.0	6.3
18. Chad	18.6	14.0	14.5	6.5
19. Togo	16.1	10.0	24.8	4.6
20. Tanzania	35.8	26.6	28.1	9.6
21. Uganda	36.0	22.9	30.8	16.9
22. Zambia	28.8	20.6	22.1	11.0
23. Zimbabwe	35.0	15.9	25.2	9.6
All countries	25.6	19.4	24.3	9.6

## Results

### Exposure to interparental violence and IPV among women

The prevalence of exposure to interparental violence in the 23 countries studied was 25.6%, with Burundi having the highest (40.3%) and Comoros recording the lowest (4.7%). The pooled prevalence of physical, emotional and sexual violence among the respondents was 19.4%, 24.3% and 9.6%, respectively. Physical and emotional violence were most prevalent among women in Sierra Leone, with each recording a prevalence of 39.0%. Exposure to sexual violence was most prevalent among women in Burundi (20.3%) (Table 2).

### Distribution of IPV across the explanatory variables

Table 3 shows the prevalence of physical, emotional and sexual violence in relation to exposure to interparental violence and covariates. The results revealed significant differences in physical, emotional and sexual violence among those who had experienced interparental violence. Physical violence was more prevalent among women who had experienced interparental violence (30.0%) than among those who had not (15.7%). Emotional violence was also higher (33.9%) among women who had experienced interparental violence than among those who had not (21.0%). Sexual violence was also more common among women who had witnessed interparental violence (15.7%) than among those who had not (7.6%). Maternal age, maternal educational level, marital status, current working status, exposure to television, exposure to newspaper or magazine, partner's age, partner's educational level, wealth index and place of residence were all associated with physical, emotional and sexual violence at p<0.05.

**Table 3.** Bivariate analysis of exposure to interparental violence and experience of IPV among women in SSA

			Physical violence		Emotional violence		Sexual violence	
Variable	Weighted N	Weighted %	Yes (%)	p	Yes (%)	p	Yes (%)	p
Exposure to interparental violence				<0.001		<0.001		<0.001
No	72 012	74.4	15.7		21.0		7.6	
Yes	24 770	25.6	30.0		33.9		15.4	
Maternal age (y)				<0.001		<0.001		<0.001
15–19	6057	6.3	19.1		20.1		10.2	
20–24	16 729	17.3	22.9		24.8		11.1	
25–29	20 795	21.5	21.6		25.7		10.3	
30–34	18 536	19.1	19.4		25.0		9.9	
35–39	15 554	16.1	17.5		24.7		9.0	
40–44	10 990	11.3	16.6		23.3		7.6	
45–49	8120	8.4	14.2		21.8		7.2	
Maternal educational level				<0.001		<0.001		<0.001
No education	31 962	33.0	17.8		23.8		8.3	
Primary	35 109	36.3	22.1		26.1		12.1	
Secondary	25 172	26.0	19.4		23.9		8.8	
Higher	4539	4.7	9.3		16.5		3.4	
Marital status				<0.001		<0.001		<0.001
Married	74 857	77.3	17.6		23.4		9.0	
Cohabiting	21 925	22.7	25.3		27.5		11.4	
Current working status				<0.001		<0.001		<0.001
No	31 178	32.2	17.6		20.8		8.4	
Yes	65 604	67.8	20.2		26.0		10.1	
Exposure to radio				0.001		0.482		0.003
No	59 502	61.5	20.1		24.4		9.9	
Yes	37 280	38.5	18.3		24.1		9.0	
Exposure to television				<0.001		<0.001		<0.001
No	69 437	71.7	20.1		25.2		10.7	
Yes	27 345	28.3	17.5		22.0		6.7	
Exposure to newspaper/magazine				0.026		<0.001		<0.001
No	88 941	91.9	19.5		24.6		9.8	
Yes	7841	8.1	17.7		21.3		7.0	
Partner's age (y)				<0.001		0.001		<0.001
15–24	6024	6.2	23.9		23.9		12.4	
25–34	30 738	31.8	22.2		24.6		10.8	
35–44	31 939	33.0	19.2		25.1		9.8	
≥45	28 081	29.0	15.6		23.2		7.3	
Partner's educational level				<0.001		<0.001		<0.001
No education	24 621	25.4	17.3		23.3		8.2	
Primary	31 445	32.5	22.0		26.2		12.1	
Secondary	31 924	33.0	20.3		24.7		9.4	
Higher	8792	9.1	12.6		18.9		4.9	
Wealth index				<0.001		<0.001		<0.001
Poorest	18 689	19.3	22.1		26.5		10.7	
Poorer	19 618	20.3	21.8		25.9		10.9	
Middle	19 488	20.1	20.2		25.2		10.2	
Richer	19 467	20.1	18.9		24.3		9.8	
Richest	19 520	20.2	14.0		19.7		6.3	
Place of residence				0.047		0.005		<0.001
Urban	34 678	35.8	18.8		23.3		7.6	
Rural	62 104	64.2	19.7		24.9		10.6	

Note: p values were generated from χ^2^ test.

### Association between exposure to interparental violence and experience of IPV among women in SSA

Table 4 shows the findings of the association between interparental violence and IPV among women in SSA. Women who had experienced interparental violence were more likely to experience physical (aOR=2.17, 95% CI 2.07 to 2.28), emotional (aOR=1.87, 95% CI 1.78 to 1.96) and sexual (aOR=2.02, 95% CI 1.90 to 2.15) violence compared to those who had not witnessed interparental violence. In all 23 countries except Comoros, we found that women who were exposed to interparental violence had a higher risk of physical and emotional violence than those who were not. Except for Liberia, Rwanda and Sierra Leone, women who had experienced interparental violence were more likely to experience sexual violence (Table 5). Concerning the covariates, the likelihood of physical, emotional and sexual violence was higher among women aged 20–34 y and those who were cohabiting relative to those aged 15–19 y and those who were married, respectively. Women with a higher level of education were less likely to experience physical, emotional and sexual violence compared to those with no formal education. Women who were working were more likely to experience physical, emotional and sexual violence than those who were not working.

**Table 4.** Multivariable regression analysis of women's exposure to interparental violence and experience of IPV among women in SSA

Variable	Physical violence	Emotional violence	Sexual violence
	aOR (95% CI)	aOR (95% CI)	aOR (95% CI)
Exposure to interparental violence			
No	1.00	1.00	1.00
Yes	2.17*** (2.07, 2.28)	1.87*** (1.78, 1.96)	2.02*** (1.90, 2.15)
Maternal age (y)			
15–19	1.00	1.00	1.00
20–24	1.36*** (1.22, 1.51)	1.32*** (1.19, 1.47)	1.18* (1.02, 1.36)
25–29	1.39*** (1.25, 1.55)	1.41*** (1.27, 1.57)	1.19* (1.03, 1.38)
30–34	1.30*** (1.16, 1.47)	1.34*** (1.19, 1.50)	1.21* (1.04, 1.42)
35–39	1.19** (1.05, 1.35)	1.28*** (1.14, 1.45)	1.13 (0.96, 1.34)
40–44	1.19* (1.03, 1.37)	1.19* (1.04, 1.36)	1.03 (0.86, 1.24)
45–49	1.03 (0.88, 1.20)	1.10 (0.95, 1.26)	1.03 (0.84, 1.26)
Maternal educational level			
No education	1.00	1.00	1.00
Primary	1.09** (1.03, 1.16)	1.03 (0.97, 1.09)	1.29*** (1.19, 1.39)
Secondary	1.05 (0.97, 1.14)	1.05 (0.98, 1.13)	1.17** (1.06, 1.30)
Higher	0.61*** (0.50, 0.76)	0.80* (0.67, 0.95)	0.64*** (0.49, 0.83)
Marital status			
Married	1.00	1.00	1.00
Cohabiting	1.35*** (1.28, 1.44)	1.15*** (1.08, 1.22)	1.19*** (1.09, 1.29)
Current working status			
No	1.00	1.00	1.00
Yes	1.19*** (1.13, 1.26)	1.31*** (1.24, 1.38)	1.17*** (1.09, 1.26)
Exposure to radio			
No	1.00	1.00	1.00
Yes	0.92** (0.87, 0.97)	1.03 (0.98, 1.08)	0.98 (0.92, 1.05)
Exposure to television			
No	1.00	1.00	1.00
Yes	0.97 (0.90, 1.04)	0.92* (0.86, 0.98)	0.72*** (0.65, 0.80)
Exposure to newspaper/magazine			
No	1.00	1.00	1.00
Yes	1.04 (0.93, 1.16)	0.91 (0.83, 1.01)	0.94 (0.81, 1.10)
Partner's age (y)			
15–24	1.00	1.00	1.00
25–34	0.92 (0.83, 1.02)	0.98 (0.88, 1.08)	0.91 (0.80, 1.04)
35–44	0.83** (0.75, 0.93)	1.04 (0.93, 1.16)	0.86* (0.74, 0.99)
≥45	0.72*** (0.63, 0.81)	1.02 (0.90, 1.15)	0.68*** (0.58, 0.80)
Partner's educational level			
No education	1.00	1.00	1.00
Primary	1.12** (1.05, 1.20)	1.05 (0.99, 1.12)	1.18*** (1.08, 1.30)
Secondary	1.07 (0.99, 1.16)	1.05 (0.98, 1.12)	1.04 (0.93, 1.16)
Higher	0.88 (0.77, 1.01)	0.93 (0.82, 1.04)	0.79* (0.65, 0.95)
Wealth index			
Poorest	1.00	1.00	1.00
Poorer	0.98 (0.92, 1.04)	0.96 (0.90, 1.02)	1.03 (0.95, 1.12)
Middle	0.87*** (0.81, 0.94)	0.92* (0.86, 0.98)	1.00 (0.91, 1.10)
Richer	0.76*** (0.70, 0.82)	0.85*** (0.78, 0.92)	1.05 (0.94, 1.17)
Richest	0.57*** (0.51, 0.63)	0.69*** (0.62, 0.76)	0.86* (0.75, 0.99)
Place of residence			
Urban	1.00	1.00	1.00
Rural	0.76*** (0.71, 0.82)	0.85*** (0.80, 0.91)	1.06 (0.96, 1.18)

* *p*<0.05; **p<0.01; ***p<0.001; 1.00=reference category.

**Table 5.** Regression analysis of women's exposure to interparental violence and experience of IPV among women in SSA by country

Country	Physical violence	Emotional violence	Sexual violence
	aOR (95% CI)	aOR (95% CI)	aOR (95% CI)
1. Angola	1.76*** (1.55, 2.01)	1.53*** (1.34, 1.75)	2.19*** (1.77, 2.70)
2. Benin	3.22*** (2.46, 4.21)	2.63*** (2.12, 3.28)	2.23*** (1.57, 3.17)
3. Burundi	1.76*** (1.55, 2.00)	1.55*** (1.36, 1.76)	1.37*** (1.21, 1.56)
4. DR Congo	1.99*** (1.75, 2.26)	2.13*** (1.87, 2.41)	1.72*** (1.49, 1.99)
5. Cameroon	2.20*** (1.83, 2.64)	1.76*** (1.47, 2.10)	1.39* (1.05, 1.85)
6. Ethiopia	2.08*** (1.72, 2.50)	2.56*** (2.15, 3.03)	1.63** (1.23, 2.16)
7. Gabon	1.80*** (1.53, 2.11)	1.74*** (1.48, 2.06)	1.91*** (1.51, 2.43)
8. Gambia	2.03*** (1.40, 2.94)	2.84*** (2.06, 3.92)	5.21*** (2.57, 10.5)
9. Kenya	1.84*** (1.56, 2.16)	1.78*** (1.52, 2.09)	2.19*** (1.73, 2.77)
10. Comoros	1.53 (0.67, 3.49)	1.60 (0.80, 3.19)	4.33** (1.51, 12.37)
11. Liberia	1.65*** (1.33, 2.05)	1.78*** (1.44, 2.20)	1.40 (0.94, 2.07)
12. Mali	3.39*** (2.57, 4.49)	2.69*** (2.06, 3.51)	2.36*** (1.60, 3.48)
13. Malawi	1.74*** (1.46, 2.07)	1.72*** (1.47, 2.00)	1.73*** (1.45, 2.05)
14. Nigeria	3.45*** (2.93, 4.06)	3.42*** (2.97, 3.95)	2.51*** (1.97, 3.20)
15. Namibia	2.07*** (1.50, 2.85)	2.03*** (1.48, 2.76)	2.67*** (1.56, 4.58)
16. Rwanda	1.61*** (1.23, 2.10)	1.41* (1.09, 1.83)	1.20 (0.84, 1.73)
17. Sierra Leone	2.68*** (2.30, 3.12)	2.15*** (1.85, 2.50)	1.32 (0.99, 1.77)
18. Chad	3.27*** (2.51, 4.26)	2.98*** (2.29, 3.88)	3.32*** (2.34, 4.71)
19. Togo	2.46*** (2.01, 3.02)	2.12*** (1.81, 2.49)	2.36*** (1.77, 3.14)
20. Tanzania	2.18*** (1.93, 2.45)	2.19*** (1.94, 2.46)	2.36*** (1.99, 2.81)
21. Uganda	1.66*** (1.48, 1.88)	1.66*** (1.49, 1.86)	1.80*** (1.57, 2.06)
22. Zambia	1.90*** (1.66, 2.17)	1.98*** (1.73, 2.25)	1.90*** (1.61, 2.25)
23. Zimbabwe	1.68*** (1.43, 1.97)	1.72*** (1.50, 1.98)	1.57*** (1.28, 1.92)

* *p*<0.05; **p<0.01; ***p<0.001; 1.00=reference category.

## Discussion

This study aimed at examining the association between exposure to interparental violence and the experience of IPV among women in SSA. While the prevalence of IPV reported in the current study is high and corresponds to a previous work,^11^ we have observed that our study reports a slightly lower prevalence of violence than the proportion of IPV in the previous study. The current study found that the prevalence of IPV in Nigeria was higher than previously reported prevalence in population-based studies, for physical (10.4%), emotional (14.3%) and sexual (2.3%) violence.^19^ The observed disparities in prevalence could be attributed to differences in study periods and the number of countries involved in the study. The trends of experience of the various forms of IPV, with emotional violence being the most common, are similar to what has been reported in South Africa, where 81% of pregnant women who experience IPV reported emotional abuse, 76% reported physical abuse and 26% reported sexual abuse.^26^ Emotional and physical violence have been identified as common forms of IPV in countries in SSA,^17,27^ and sexual violence appears to be under-reported in several African settings because of the stigma associated with IPV victims.^17^

The prevalence of women's exposure to interparental violence in this study is similar to the prevalence reported in some developing countries, such as Bangladesh (26.4%),^18^ but significantly higher than the prevalence reported in many developed countries (e.g. France [21%]).^28^ The high prevalence of interparental violence in SSA suggests that many people may be exposed to interparental violence at some point in time in their childhood and/or lifetime.

We found that exposure to interparental violence is associated with women experiencing physical, emotional and sexual violence. This finding is consistent with the findings of similar studies conducted in Nigeria^19,20^ and South Africa,^29^ which found that women who had witnessed interparental violence were more likely to experience physical, emotional and sexual violence than those who were not exposed. The significant relationship between interparental violence and IPV in this study reflects the need to consider interparental violence as an important component in interventions being developed to reduce IPV in SSA. Violence is widely regarded as a learned behavior that can be passed down from generation to generation.^18^ According to the Social Learning Theory,^30^ learning and intensification of new behaviors can be accomplished or undone by observing or modeling significant others. The emotional and behavioral development of children are indicated to be influenced mainly by their parents as the main agents for making them fit into society (i.e. socialization),^18^ therefore, children acquire behaviors and attitudes by observing their parents and emulating their behavior.^18^ For that matter, children observing the perpetration and victimization of their parents may subconsciously acquire similar experiences, especially in situations where the perpetration of IPV is done with impunity. Consequently, IPV may be seen by children as the exertion of authority and an acceptable behavior, which may lead to the replication of such behaviors. For instance, males exposed to interparental violence where their fathers are the perpetrators of violence may see violence against women as the norm later in life, whereas women exposed to interparental violence where their mothers were the victims may tend to tolerate IPV.^20^ Several studies have established that exposure to interparental violence is associated with attitudes of tolerating or justifying IPV and victimization in women^19^ and the perpetration of IPV by men.^3^ Findings from the current study suggest the need for both preventive and reactive interventions that can mitigate against both the exposure of children to interparental violence (preventive) and the current experience of IPV (reactive) in sexual unions. Such interventions can help to break the cycle of violence in many vulnerable homes and subsequently limit the intergenerational transmission of IPV.

### Strengths and limitations

The current study presented evidence that supports the importance of understanding the role played by interparental violence in the victimization of IPV among women in SSA, which could have important implications for health policy and IPV interventions in the region. The study also relied on relatively large amounts of data from nationally representative samples from 23 countries, which improves the accuracy and generalizability of the findings.

However, the study's findings are limited in some ways. First, because the study relied on cross-sectional data, causal interpretations of the findings are limited. Second, the study relied on self-reported data that could not be independently verified, so the prevalence of IPV and exposure to interparental violence could be underestimated or overestimated. Additionally, the data for the study were limited to only women, which conforms to the contestable notion of gender bias in the report of a vulnerability in matters of IPV.

## Conclusions and implications

The study has established a strong relationship between exposure to interparental violence and IPV across several countries in the sub-Saharan African region. Our finding underscores the need for policies and programs aimed at addressing the problems of IPV to pay particular attention to children being exposed to interparental violence. Interventions could be targeted at identifying children exposed to interparental violence and giving them support to cope with the exposures and reduce behavioral problems and the subsequent risk of IPV in their adulthood. Families at risk of IPV, such as those with a history of interparental violence, could be supported through counseling and by increasing the awareness of the negative consequences of IPV to help deal with their learned experiences.

## Data Availability

The dataset is freely accessible via this link: https://dhsprogram.com/data/available-datasets.cfm.
